# The influence of footwear on walking biomechanics in individuals with chronic ankle instability

**DOI:** 10.1371/journal.pone.0239621

**Published:** 2020-09-24

**Authors:** Gabriel Moisan, Martin Descarreaux, Vincent Cantin

**Affiliations:** 1 Department of Anatomy, Université du Québec à Trois-Rivières, Trois-Rivières, PQ, Canada; 2 Department of Human Kinetics, Université du Québec à Trois-Rivières, Trois-Rivières, PQ, Canada; 3 Groupe de recherche sur les affections neuro-musculo-squelettiques (GRAN), Université du Québec à Trois-Rivières, Trois-Rivières, PQ, Canada; São Paulo State University (UNESP), BRAZIL

## Abstract

**Background/Purpose:**

The effects of footwear on the walking kinematics, kinetics and electromyography (EMG) of individuals with chronic ankle instability (CAI) at different speeds are still unknown. The objective of this cross-sectional study was to evaluate the kinematic, kinetic and electromyography differences between shod and barefoot walking at comfortable (CW) and fast (FW) speeds in individuals with CAI.

**Methods:**

Twenty-one individuals with CAI walked on a 5-meter walkway shod and barefoot at CW and FW speeds. A force plate was used to record the ground reaction forces, a 3-D motion analysis system to record the lower limb kinematics and a surface EMG system to collect the gluteus medius, vastus lateralis, gastrocnemius lateralis, gastrocnemius medialis, peroneus longus and tibialis anterior muscles activity. The dependent variables were ankle and knee angles and moments and normalized muscle activity. The shod and barefoot data during CW and FW were compared using a one-dimensional non-parametric mapping analysis.

**Results:**

The main results of this study were that individuals with CAI exhibited more ankle dorsiflexion angle, knee extension and tibialis anterior muscle activation during the beginning of the stance phase during shod compared to barefoot walking. Also, the biomechanical effects of shoes are similar during walking at FW and CW.

**Conclusion:**

The biomechanical deficits associated with CAI were partly attenuated during the shod compared to the barefoot condition and these effects were similar at CW and FW. These findings are compatible with the concept that locomotor interventions using suitable shoes may enhance gait abilities in individuals with CAI.

## Introduction

Shoes are important in human locomotion, as they are the first interface between the body and the ground. Shoes are mainly worn to protect the feet from thermal and/or chemical injuries, to enhance daily comfort but also to modulate lower limbs’ biomechanics. During shod walking, cadence [1], ankle plantarflexion [2], knee flexion [3] and knee flexion moments [4] are decreased and knee adduction moments [4], tibialis anterior muscle activity [5], peroneus longus muscle activity [5] and stride length [4] are increased compared to barefoot walking. Changes in lower limb biomechanics when wearing shoes have been associated with improvement in pain and function in individuals with musculoskeletal disorders [6, 7]. Individuals with chronic ankle instability (CAI) could perhaps benefit from the biomechanical effects of wearing certain types of shoes, such as the decrease in ankle plantarflexion. CAI is characterized by a continuum of residual impairments following a lateral ankle sprain and results in recurrent sprains and/or episodes of ankle “giving way” during locomotion [8]. During walking, individuals with CAI exhibit increased ankle and rearfoot inversion, ankle plantarflexion, lateral foot vertical forces and peroneus longus muscle activity compared to healthy controls [9]. However, no previous study simultaneously identified the biomechanical differences between individuals with and without CAI when walking shod and barefoot. Also, no study has quantified lower limb biomechanical effects of wearing shoes during walking in individuals with CAI. It has yet to be determined if biomechanical deficits associated with CAI can be attenuated by wearing shoes. Quantifying the biomechanical effects of shoes on individuals with CAI will help clinicians and researchers to better understand their potential benefits in the rehabilitation of this population.

In most studies quantifying the lower limb biomechanical effects of shoes, participants were asked to walk at a self-selected, comfortable speed, as shown by a recent systematic review [10]. Walking at a faster speed increases lower extremity muscle activity [11], joint moments [12], tibio-talar plantarflexion and hallux dorsiflexion at toe off [13]. As lower limb biomechanics change as walking speed increases, shoes could induce different biomechanical effects at different speeds. There is a need to investigate the effects of shoes on lower limb biomechanics when walking at a faster speed in order to better understand the role of footwear during locomotion.

The objectives of this study were to quantify the effects of wearing shoes on lower limb EMG, kinematics and kinetics in individuals with CAI during walking and to assess if these effects change at a faster speed. It was hypothesized that wearing shoes will decrease the biomechanical deficits associated with CAI, such as the increased ankle plantarflexion, and that the effects will be greater when walking faster.

## Materials and methods

### Participants

Twenty-one individuals with CAI were recruited to participate in this cross-sectional study (see Table 1). Participants were identical to the CAI group of a study investigating the biomechanical differences between individuals with and without CAI during shod walking [14]. Participants were included if they had one (or more) significant lateral ankle sprains that occurred more than one year prior to study onset and had self-reported functional deficits due to ankle symptoms that were quantified by a score of <90% and <80% on the Foot and Ankle Ability Measure (FAAM) Activity of daily living (ADL) and Sport (S) subscales, respectively. At least two episodes of an ankle “giving way” in the last six months and/or having a feeling of instability had to be reported by the participants in order to be included in this study. Individuals were excluded from the study if they had a history of a lower extremity surgery or a fracture that needed a surgical realignment, a history of lower extremity musculoskeletal injury within the last three months, had any known condition that adversely affects gait or were undergoing treatment for CAI. Inclusion criteria were based on the recommendations of the International Ankle Consortium [15]. The Université du Québec à Trois-Rivières (UQTR) ethics committee (CER-16-226-07.21) approved the experimental protocol, and written consent was obtained from each participant. The potential participants were recruited through the university’s outpatient podiatry clinic and among the UQTR students.

**Table 1:** Descriptive data.

Gender ratio (M/F)	4/17
Age (years)	26.3 (±8.5)
Mass (kg)	64.9 (±12.7)
Height (m)	1.65 (± 0.08)
Foot posture index	3.3 (±3.8)
Last sprain (yr)	2.4 (±1.9)
Previous sprains	5.6 (±5.4)
FAAM-ADL (%)	86.4 (±4.5)
FAAM-Sport (%)	69.6 (±8)
IPAQ (MET-min/week)	2125 (±1468)

Data reported as mean (standard deviation).

### Instrumentation

Kinematic markers were placed on the tested limb over the greater trochanter, the lateral femoral epicondyle, the lateral malleolus and the fifth metatarsal head. Two three-marker clusters were placed on the distal lateral one-third of the leg and thigh. During the shod condition, the marker over the fifth metatarsal head was attached directly to the shoe as determined by palpation. For the sake of ecological validity, it was chosen not to cut holes into the shoe uppers in order not to decrease their stability. Also, a limitation of the data collection volume area was associated with the motion analysis system used. It was therefore not feasible to reliably place multiple markers on the foot in a non-colinear way. During a calibration trial, virtual markers were created over the medial femoral epicondyle and medial malleolus. The calibration trial was used to locate the hip/knee/ankle joint centers and subsequently calculate ankle and knee angles and moments during the dynamic trials. Nine Optotrak Certus cameras (Northern Digital, Waterloo, Ontario, Canada) recording at a sampling rate of 100 Hz, collected the kinematic data.

To collect ground reaction forces data, a force platform (Bertec Corp, Colombus, OH, USA) embedded in the floor on the participants’ path was used at a sampling rate of 1000 Hz. To record walking speed, electronic photocells timing gates (Brower Timing System, Draper, UT, USA) positioned 1.35 meters before and after the force platform were used.

Differential Ag surface EMG electrodes (Model DE2.1, Delsys Inc, Boston, MA, USA) were placed over the gluteus medius, vastus lateralis, gastrocnemius lateralis and medialis, peroneus longus and tibialis anterior muscles in the position outlined by the SENIAM guidelines [16]. At each electrode site, the skin was shaved, abraded with fine-grade sandpaper and wiped with alcohol swabs. A reference electrode was placed over the ipsilateral anterior superior iliac spine. The EMG signals were collected at a sampling rate of 1000 Hz and amplified with a gain of 1000 (CMRR > 92 dB at 60 Hz, input impedance of 10 GW; 12 bits A/D converter).

### Protocol

Prior to the experimental protocol, the participants completed the validated French version of the FAAM-ADL and FAAM-S [17] and the International Physical Activity Questionnaire (IPAQ) [18]. They also reported the number of sustained ankle sprains and the time since the last sprain. In order to quantify foot morphology of the participants, the Foot Posture Index (FPI) was administered to them [19].

Prior to the data collection, a calibration trial was recorded. Then, participants had to walk on a 5-meter walkway at self-selected comfortable (CW) and fast (FW) speeds during two experimental conditions (shod and barefoot). The order of the conditions and speeds was randomly assigned across participants. The FW was described to the participants as the fastest walking speed possible to reach whilst keeping both feet simultaneously in contact with the ground. During the shod condition trials, all participants wore the same model of shoes, in the proper size (Athletic Works, Model: Rupert, Bentonville, AR, USA). The protocol consisted of completing five familiarization trials during which mean walking speed was calculated and five trials during which lower limb kinetics, kinematics and EMG was recorded. It was performed for each condition and speed for 20 consecutive recorded trials. A trial was rejected and immediately retaken when speed varied from ±5% of the mean speed determined during the familiarization trials, if the foot was not entirely on the force platform, or if participants adapted their stride length or frequency in an attempt to hit the force platform.

#### Data processing

Kinematic and kinetic data were processed using Visual3D software (C-motion, Inc., Germantown, MD, USA). The dependent variables were ankle and knee angles and moments. Before joint angle and moment calculations, marker trajectories and force platform data were respectively low-pass filtered using a 4th order Butterworth filter at a frequency of 6 Hz and 50 Hz. Interjoint motion was calculated using a Cardan sequence of X-Y’-Z”. Rotation around the X, Y’ and Z”-axes defined the extension/flexion, adduction/abduction and internal rotation/external rotation, respectively. All movements were expressed as rotation of the distal segment in relation to the proximal segment. The kinematic model used in this study only allowed measuring the sagittal plane angle and moment (X) of the ankle joint. The calibration trial ankle angle was determined as the 0° of the joint. The knee and ankle joints centers were calculated as the midpoint from the medial-lateral aspect of each joint. Internal joint moments at the knee and ankle were calculated using inverse dynamics and were normalized to body mass (Nm/kg). Touchdown and toe-off of the stance phase were determined with the force platform using a 10 N threshold.

EMG data of all muscles were filtered with a 10 to 450 Hz 4th order Butterworth band-pass filter using a custom MATLAB file (Mathworks, Inc., Natick, MA). The Root Mean Square (RMS) of these data was calculated with a 100 ms-moving window. The RMS data of all trials were normalized with the mean peak RMS of the five shod trials at fast walking speed.

### Analysis

Walking speeds during shod and barefoot trials at CW and at FW were compared with dependent t tests as the data were distributed according to the Shapiro-Wilk test. To compare the EMG, kinematic and kinetic data between shod and barefoot walking during CW and FW, curves analyses were performed using one-dimensional statistical non-parametric mapping (SnPM) [20, 21]. Each individual stance phase was normalized to 100%. A Bonferroni threshold of significance of *P*<0.0031 (*P* = 0.05/16) was used to correct for multiple comparisons. When the SnPM(t) curves crossed this threshold for the biomechanical outcomes, supra-threshold clusters were created, indicating significant differences between the shod and barefoot conditions in a specific location of the stance phase. The analyses were conducted using the open-source code (www.spm1d.org) in Python software (Version 2.7).

## Results

### Walking speed

During shod trials at FW, mean walking speed was faster compared to barefoot trials (2.00 m/s (SD:0.23) vs 1.89 m/s (SD:0.29) (*P*<0.001)). No difference was observed between conditions at CW (1.38 m/s (SD:0.19) vs 1.32 m/s (SD:0.21) (*P* = 0.12)).

### Kinematic and kinetic data

**Fig 1:**
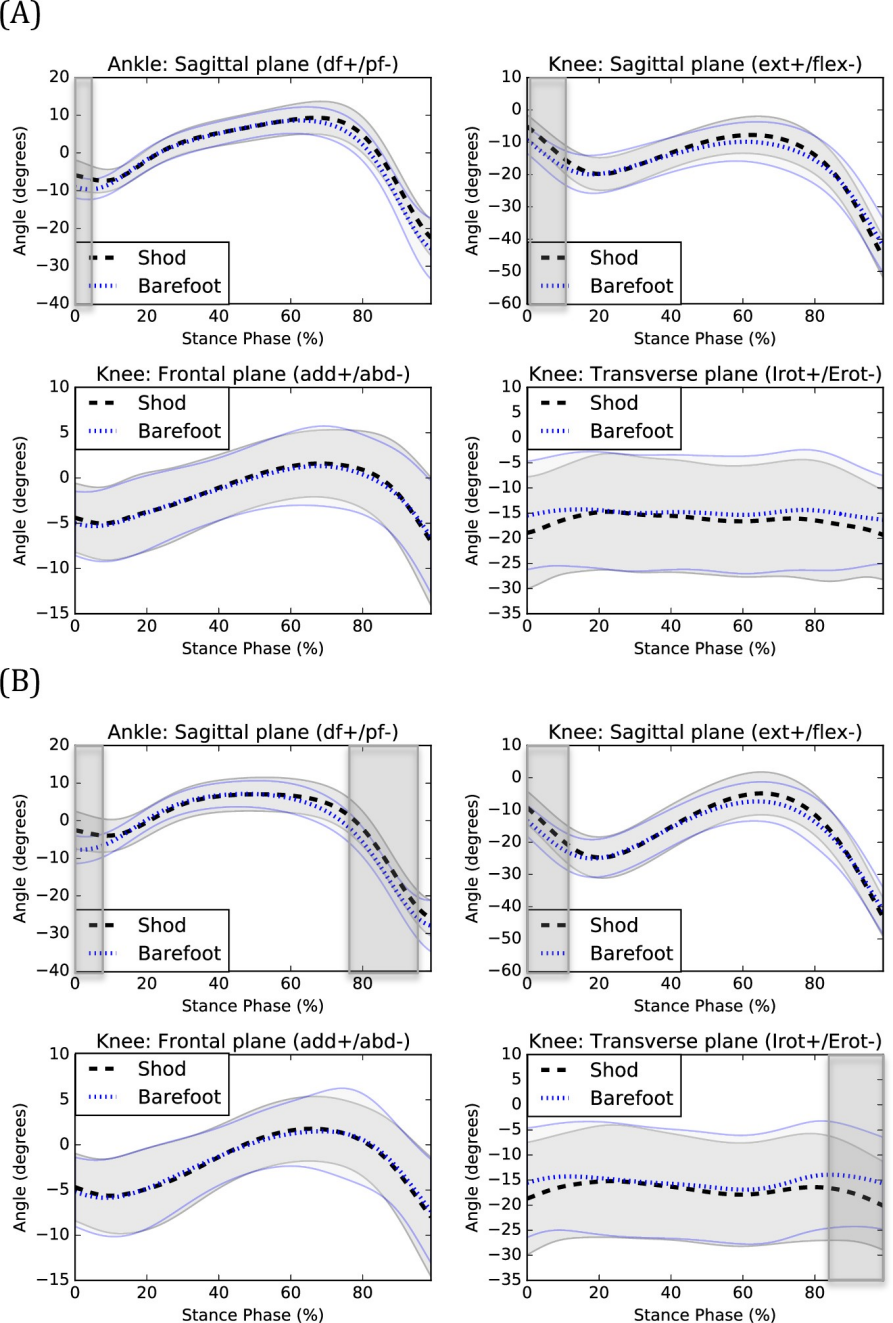
Kinematics of shod and barefoot walking during (A) CW and (B) FW. Means of the shod (black) and barefoot (blue) conditions are respectively represented by dotted lines and standard deviations are observed between the full lines. Significant between-group differences are observed in the shadowed region. df: dorsiflexion, pf: plantarflexion, ext: extension, flex: flexion, add: adduction, abd: abduction, Irot: internal rotation, Erot: external rotation.

**Fig 2:**
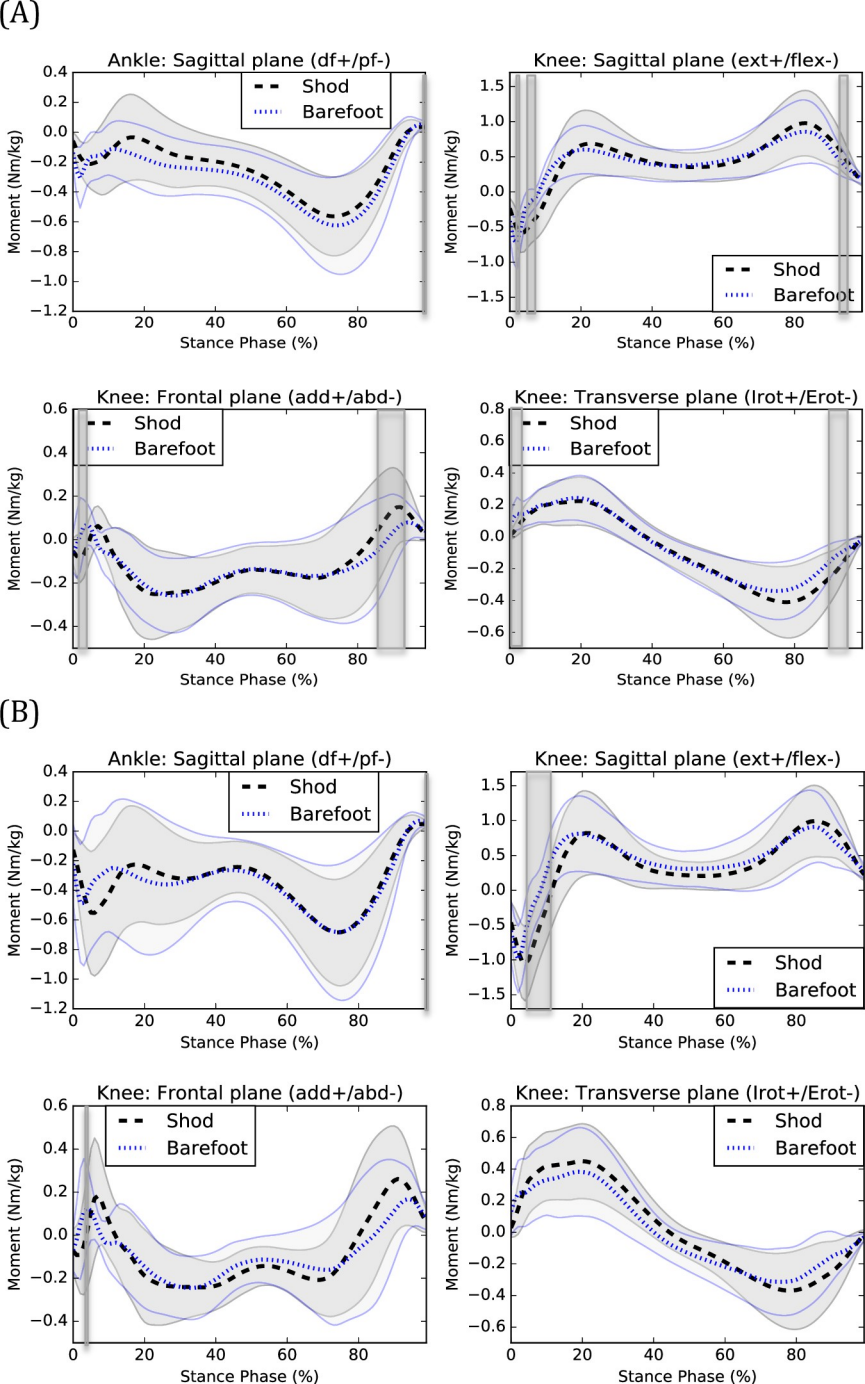
Kinetics of shod and barefoot walking during (A) CW and (B) FW. Means of the shod (black) and barefoot (blue) conditions are respectively represented by dotted lines and standard deviations are observed between the full lines. Significant between-group differences are observed in the shadowed region. df: dorsiflexion, pf: plantarflexion, ext: extension, flex: flexion, add: adduction, abd: abduction, Irot: internal rotation, Erot: external rotation.

During the data collection session of one participant, technical difficulties occurred and thus this dataset was removed from the kinematic and kinetic analyses. Graphical representations of kinematic and kinetic patterns are presented in Fig 1 and Fig 2.

During shod CW, participants exhibited increased ankle dorsiflexion angle from 0 to 5% (*P* = 0.001) and decreased dorsiflexion moments from 98 to 99% (*P* = 0.001) of the stance phase compared to barefoot walking. They also exhibited increased knee extension angle from 0 to 11% (*P*<0.001), adduction moments from 85 to 93% (*P*<0.001), extension moments at 1% (*P* = 0.001) and from 91 to 94% (*P*<0.001) and decreased extension moments from 4 to 7% (*P*<0.001), adduction moments from 2 to 4% (*P*<0.001) and internal rotation moments from 0 to 3% (*P*<0.001) and 89 to 94% (*P* = 0.001) of the stance phase during shod compared to barefoot walking.

During shod FW, participants exhibited increased ankle dorsiflexion angle from 0 to 8% (*P*<0.001) and 84 to 99% (*P*<0.001) and decreased dorsiflexion moments at 99% (*P* = 0.001) of the stance phase for shod compared to barefoot walking. At the knee, increased extension angle from 0 to 13% (*P*<0.001) and decreased internal rotation angle from 84 to 99% (P<0.001), extension moments from 4 to 12% (*P*<0.001), adduction moments at 3% (*P* = 0.001) of the stance phase were observed during shod compared to barefoot walking.

### EMG data

Technical difficulties with the EMG measurements led to remove a few datasets (e.g. excessive perspiration). The following numbers of participants were used in the EMG analyses of the gluteus medius (20 CW, 21 FW), vastus lateralis (20 CW, 21 FW), gastrocnemius lateralis (19 CW, 19 FW), gastrocnemius medialis (20 CW, 21 FW), peroneus longus (19 CW, 20 FW) and tibialis anterior (20 CW, 21 FW) muscles.

At CW, tibialis anterior muscle activity was increased from 0 to 1% (*P* = 0.001) and 4 to 12% (*P*<0.001) of the stance phase during shod compared to barefoot walking.

**Fig 3:**
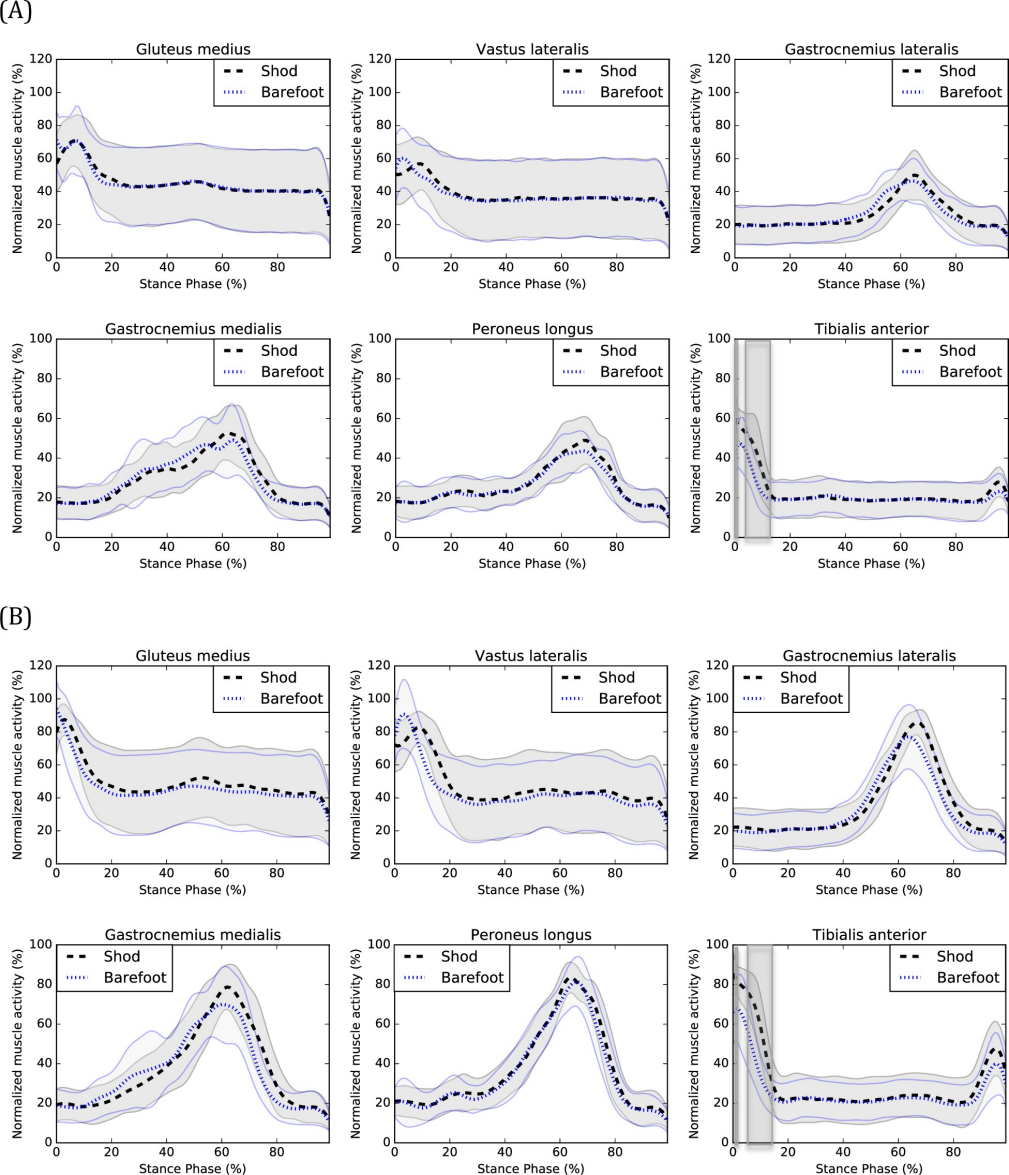
EMG of shod and barefoot walking during (A) CW and (B) FW. Means of the shod (black) and barefoot (blue) conditions are respectively represented by dotted lines and standard deviations are observed between the full lines. Significant between-group differences are observed in the shadowed region.

At FW, muscle activity was increased for the vastus lateralis from 10 to 17% (*P*<0.001), the gastrocnemius lateralis from 68 to 82% (*P*<0.001), the gastrocnemius medialis from 73–78% (*P*<0.001) and the tibialis anterior from 0 to 1% (*P* = 0.001) and 5 to 14% (*P*<0.001) of the stance phase during shod compared to barefoot walking. Graphical representations of EMG patterns are presented in Fig 3.

## Discussion

Individuals with CAI exhibited changes in EMG, kinetics and kinematics during shod compared to barefoot walking. The main kinematic effect of wearing shoes was increased ankle dorsiflexion during the beginning of the stance phase (CW and FW). Previous studies showed that individuals with CAI exhibit decreased ankle dorsiflexion (or increased plantarflexion) compared to healthy counterparts during walking [22, 23]. The posterior part of the talar trochlea is narrower than the anterior part [24]. When the ankle joint is in a dorsiflexed position, the anterior part of the talar trochlea is in contact with the articular surfaces of the malleoli, decreasing the talo-crural space width [25] and increasing ankle stability [26]. Therefore, the decreased ankle dorsiflexion during walking could place individuals with CAI at more risk of sustaining a lateral ankle sprain [8]. As most lateral ankle sprains are sustained during the initial impact of the foot with the ground during locomotion [27–29], wearing shoes could perhaps attenuate the risk of sustaining lateral ankle sprains during walking. In fact, in our study, most shoes’ significant effects were observed during the beginning of the stance phase. Prospective studies are needed to determine to what extent wearing shoes can decrease the risk of sustaining lateral ankle sprains and what types of shoes are the most efficient. This is especially important as previous studies showed that individuals with CAI still exhibit biomechanical deficits compared to healthy counterparts during shod walking [9]. The results of our study are consistent with those of Oeffinger et al. [2] which also observed increased ankle dorsiflexion when wearing shoes during walking. Furthermore, the increased knee extension during the beginning of the stance phase found in our study is consistent with the results of Zhang et al. [3]. This could represent a compensation for the increased ankle dorsiflexion and may be associated with the increased stride length when wearing shoes [4]. The combination of the increased ankle dorsiflexion and knee extension are also observed when comparing shod and barefoot running [30].

Knee adduction moments were decreased from 2 to 4% (CW) and at 3% (FW) of the stance phase of walking and knee extension moments were increased at 1% (CW) and decreased from 4 to 7% (CW) and 4 to 12% (FW) of the stance phase. However, when visually inspecting the knee frontal and sagittal moments curves (see Fig 2), one can observe a temporal delay suggesting that wearing shoes delays the peak knee adduction and flexion moments. It is also observed for the vastus lateralis muscle activation (FW) (see Fig 3), suggesting that wearing shoes delays the activation of this muscle which is consistent with previously published results [31]. This delayed vastus lateralis activation could perhaps be explained by the delayed peak knee adduction and flexion moments.

An increased activity of the gastrocnemius medialis and lateralis muscles (FW) was also observed during the end of the stance phase, which is consistent with the results of Franklin et al. [5], which also found an increased activity of the gastrocnemius medialis muscle during the latter part of the stance phase when wearing shoes. The increased activity occurred shortly after the peak amplitude during the beginning of the propulsion phase of walking. One of the main functions of the gastrocnemius medialis and lateralis muscles is to facilitate the anterior progression of the center of pressure and prevent the center of mass of dropping too low during the propulsive phase [32]. When walking with shoes, individuals with CAI may need a greater contribution of the gastrocnemii muscles for the propulsion to be efficient. Finally, increased tibialis anterior muscle activity was observed when wearing shoes during the beginning of the stance phase. This result is consistent with those of previous studies [5, 33] and could be responsible for the increased ankle dorsiflexion during the beginning of the stance phase, observed when wearing shoes.

Another interesting finding is that the biomechanical effects of shoes are similar during walking at FW and CW. This could be of interests for clinicians and researchers as it increases the generalizability of the previous studies’ results that investigated the biomechanical effects of shoes during walking, even if speed varied.

The results of this study should be interpreted in light of a few limitations. The first limitation is the population mean age of 26.1 years. As gait biomechanics change when getting older [34], the results of this study may not be generalizable to an older population. The second limitation is the unbalanced men/women ratio among participants. Many sex biomechanical differences have been previously observed in previous studies [35, 36]. As only four men were recruited in this study, the results may perhaps not be generalizable for the male population. The third limitation is the kinematic model used. In order to increase the ecological validity of our results, no hole was cut in the shoes upper. Therefore, only the sagittal ankle angle and moment were calculated. It is possible that shoes have significant effect on transverse and frontal ankle angles and moments but could not be observed in this study. The kinematic model also did not allow the measurement of the kinematics of the rearfoot, midfoot and forefoot segments. The fourth limitation is the greater mean speed for shod compared to barefoot walking trials at FW. The differences observed in this study represent a 5% increase, which fall into the current gold standard of ±5% in the literature. We are therefore confident that our results were not significantly biased by walking speed.

## Conclusions

The biomechanical deficits associated with CAI were partly attenuated during the shod compared to the barefoot condition and these effects were similar at CW and FW. The main results were that individuals with CAI showed more ankle dorsiflexion angle, knee extension and tibialis anterior muscle activation and delayed peak knee flexion and adduction moments during shod compared to barefoot walking. These findings are compatible with the concept that locomotor interventions using suitable shoes may enhance gait abilities in individuals with CAI. Thus, this study will inform future efficacy trials aiming to attenuate the deficits associated with CAI during rehabilitation.
